# An algorithm to detect and communicate the differences in computational models describing biological systems

**DOI:** 10.1093/bioinformatics/btv484

**Published:** 2015-10-21

**Authors:** Martin Scharm, Olaf Wolkenhauer, Dagmar Waltemath

**Affiliations:** ^1^Department of Systems Biology and Bioinformatics, University of Rostock, Rostock, Germany and; ^2^Stellenbosch Institute for Advanced Study, Wallenberg Research Centre at Stellenbosch University, Stellenbosch, South Africa

## Abstract

**Motivation:** Repositories support the reuse of models and ensure transparency about results in publications linked to those models. With thousands of models available in repositories, such as the BioModels database or the Physiome Model Repository, a framework to track the differences between models and their versions is essential to compare and combine models. Difference detection not only allows users to study the history of models but also helps in the detection of errors and inconsistencies. Existing repositories lack algorithms to track a model’s development over time.

**Results:** Focusing on SBML and CellML, we present an algorithm to accurately detect and describe differences between coexisting versions of a model with respect to (i) the models’ encoding, (ii) the structure of biological networks and (iii) mathematical expressions. This algorithm is implemented in a comprehensive and open source library called BiVeS. BiVeS helps to identify and characterize changes in computational models and thereby contributes to the documentation of a model’s history. Our work facilitates the reuse and extension of existing models and supports collaborative modelling. Finally, it contributes to better reproducibility of modelling results and to the challenge of model provenance.

**Availability and implementation:** The workflow described in this article is implemented in BiVeS. BiVeS is freely available as source code and binary from sems.uni-rostock.de. The web interface BudHat demonstrates the capabilities of BiVeS at budhat.sems.uni-rostock.de.

**Contact:** martin.scharm@uni-rostock.de

**Supplementary information:** Supplementary data are available at *Bioinformatics* online.

## 1 Introduction

Modelling and simulation is a standard approach to investigate complex biological processes. A steadily increasing number of computational models are available from open repositories such as the BioModels database (Li *et* *al.*, 2010) or the Physiome Model Repository (PMR2; Yu *et* *al.*, 2011). These repositories provide the infrastructure necessary to maintain model code and associated metadata. The distribution of models through these repositories accelerates collaborative research and encourages model reuse. The reusability of models improves the modelling workflow, by reducing errors and saving time. Tracking the evolution of a model, that is providing information about changes in the model and its encoding, plays an important role in supporting the user (Waltemath *et* *al.*, 2013a). The need of model version control has been emphasized repeatedly on several occasions (Li *et* *al.*, 2010; Miller *et* *al.*, 2011; Saffrey and Orton, 2009; Waltemath *et* *al.*, 2013a).

**Fig. 1.**
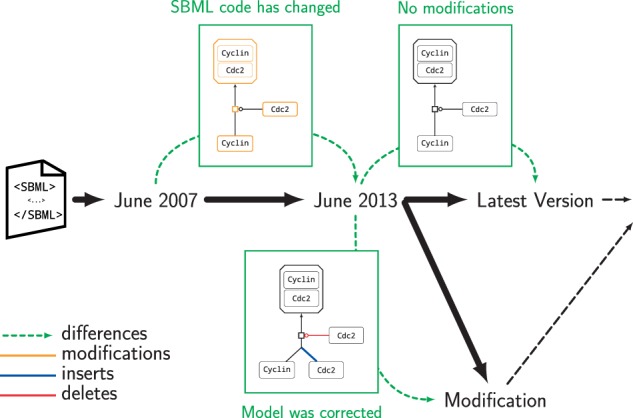
**Sketch of a model’s temporal evolution.** Changes in a single reaction of Novak and Tyson’s model with ID BIOMD0000000107 in the BioModels database. The differences between versions from June 2007 (release number 8), June 2013 (release number 25) and February 2015 (latest available version) are shown. The branch represents a modification. The boxes visualize the differences between related versions

Figure 1 visualizes the evolution of a single model. In 1993, Novak and Tyson published the first cell cycle model describing the M-phase control in *Xenopus oocyte* extracts and intact embryos (Novak and Tyson, 1993). The model representing these findings was first published in the BioModels database in 2007 (release number 8) with the identifier BIOMD0000000107 (identifiers.org/biomodels.db/BIOMD0000000107). Along with 20 official releases of the database, the model has undergone numerous changes. Figure 1 shows the differences in one of the model’s reactions, namely the formation of Cdc2-cyclin dimers from Cyclin subunits and free Cdc2 monomers (Step 3 Novak and Tyson, 1993, Fig. 1).

The relation between versions of a model from different releases can be considered the *history* of the model’s encoding (Fig. 1). In the simplest case, the history follows a single line along the time axis. In practice, however, models are subject to modifications, including corrections, extension and other refinements. After publication, models may be combined into larger networks (e.g. Gennari *et* *al*., 2008; Krause *et al.* 2010; Smallbone *et* *al*., 2010). Also during model development, several alternatives are tested, leading to different paths in the history of a model. These so-called branches complexify the study of a model’s history, especially when branches with different modifications shall be merged back into a single version of the model at a later time. For this reason, *difference detection* plays a key role in model version control. We refer to *model provenance* as the field of research that investigates the nature of differences in model versions, seeking answers to the seven W-questions: Who, What, Where, Why, When, Which, With (How)? (Davidson and Freire, 2008; Goble, 2002; Moreau *et al.*, 2008; Ram and Liu, 2010).

In this article, we present a novel algorithm for difference detection in models of biological systems. Our algorithm pre-processes the model documents, maps the hierarchical model structures and post-processes the resulting mappings. The mapping can then be exported in both machine and human readable formats. The algorithm is implemented in a software library called BiVeS. It can immediately be used with existing model repositories and model management platforms. In addition, we showcase the capabilities of BiVeS in our web-based tool BudHat for version control of models encoded in the *Systems Biology Markup Language* (SBML, Hucka *et* *al.*, 2003) or in CellML (Cuellar *et* *al.*, 2003). BiVeS and BudHat demonstrate how our work contributes to successful provenance of model-driven research in computational biology.

In the following sections, we will describe the algorithm (Section 2) in technical detail, present the results using a representative example (Section 3) and discuss the outcome and the impact of our work (Section 4).

## 2 Methods

Our algorithm for detection and communication of differences compares two versions of an XML-encoded model. It distinguishes six major steps: (Section 2.1) Pre-processing the XML documents, (Section 2.2) Mapping the hierarchical structures and (Section 2.3) Post-processing the resulting mapping. Based on the identified mapping, the algorithm computes a delta (Section 2.4). This delta can be converted into both machine readable (2.5) and human readable (2.6) formats. All steps are described in full technical detail in the following sections. Sections 2.1, 2.2 and 2.4 follow the original ideas of the XyDiff (leo.saclay.inria.fr/software/XyDiff/cdrom/www/xydiff/index-eng.htm) algorithm, which was developed at INRIA and focuses on efficiency in terms of speed and memory (Cobena *et* *al.*, 2002). The Section 3 and the Supplementary Material provide illustrative examples.

### 2.1 Pre-processing the XML documents

First, two versions of an XML-encoded model are translated into an internal tree structure. For every node *n* in the tree, a hash sum $n_{\sigma }$ and a weight $n_{\omega }$ are calculated. With length (n) denoting the length of the text stored in *n*, the weight $n_{\omega }$ is determined as in the original XyDiff algorithm:

$$n_{\omega }=\{\begin{array}{ll} 1+\log(\text{length}(n)) & \;\text{if}\;n\;\text{is}\;\text{text}\;\text{node},\;\\ 1 & \;\text{if}\;n\;\text{is}\;\text{leaf},\\ 1+\sum_{c\;\in \;\text{children}(n)}c_{\omega } & \text{otherwise}. \end{array}$$

The weight of a node is thus always greater than the weight of its children. As such, the weight represents the size of the corresponding subtree. The hash sum of a node *n* represents the signature of the subtree rooted at *n.* In the current version of our implementation, we determine the hash $n_{\sigma }$ of a node *n* by the SHA-2 sum of the concatenation of the node’s tag name, its attributes and the hash sum of all its children. While $n_{\sigma }$ unambiguously defines the subtree rooted in *n*, $n_{\sigma }$ does not need to be unique among all nodes in the tree. Thus, if $n_{\sigma }=m_{\sigma }$ then the subtrees in *n* and *m* are identically equal. We explain in the following section how these signatures speed up the mapping of two hierarchical structures.

### 2.2 Mapping the hierarchical structures

To compare two tree structures *T*_1_ and *T*_2_,__ we use XyDiff’s BULD algorithm, in which matchings are propagated bottom-up and only lazily down. It finds matchings between common large subtrees of the two documents and propagates these matchings (Cobena *et* *al.*, 2002). We distinguish four phases of mapping.

#### 2.2.1 Mapping by ID

First, nodes are being mapped with respect to their identifiers. As suggested in the original algorithm, the id attributes in the XML documents serve as identifiers. In addition, we also evaluate biological identifiers, specifically links into bio-ontologies. In our current implementation, we assign a higher priority to biological identifiers than to id attributes. In this step, nodes in both documents which share the same identifier are mapped onto each other. If many nodes are labelled with an id attribute, then a large number of mappings are already computed at this stage. Consequently, the following mapping procedure, which is computationally harder, typically simplifies.

#### 2.2.2 Bottom-up propagation

Second, the initial mapping is propagated upwards into the trees. The connections of a node’s children are evaluated in a depth-first traversal of *T*_2_. If a node *n* in *T*_2_ is connected to a node *m* in *T*_1_ then a mapping of parent(n) to parent(m) is suggested. The confidence equals $n_{\omega }$ and is therefore proportional to the size of *n*’s subtree. If, in contrast, *n* is not connected, we examine the candidates that were previously suggested by the connections of *n*’s children. Candidates which have a different tag name than *n* and candidates which already have a connection are neglected. Among the remaining candidates, the algorithm chooses the one that received the best suggestions and connects it to *n.*

#### 2.2.3 Top-down propagation

Third, the algorithm makes use of the initially computed signatures and maps nodes of *T*_2_ on nodes of *T*_1_ which share the same hash value. A priority queue Φ is maintained to sort the nodes of *T*_2_ based on their weights. Initially, Φ only consists of the root node of *T*_2_. Unless Φ is empty, the algorithm repeatedly removes node $n\in \;\Phi \subset T_{2}$ with the biggest weight, which represents the biggest subtree in the queue and collects a set of mapping candidates $M\subset T_{1}$ with $\forall m\in M:m_{\sigma }=n_{\sigma }$. If *M* is empty, all children of *n* are added to Φ and the loop continues with the next biggest subtree. Otherwise, the algorithm tries to find a node m∈M for which there already exists a mapping between ancestors(m) and ancestors(n). As proposed by Cobena *et* *al.* (2002), the number of levels to chase the ancestry of both nodes depends on the ratio of $n_{\omega }$ to $\text{root}{\left(T_{2}\right)}_{\omega }$. Thus, for large subtrees, we are willing to climb many levels in the tree to find a mapping of ancestors, but we might just examine firsthand parents to map leaf nodes. If we are able to find such an m∈M, all nodes of the subtrees in *m* and *n* are mapped onto each other, just as the ancestors up to the discovered mapping.

#### 2.2.4 Optimization

Fourth, the algorithm improves the quality of the mapping by examining the network structure of *T*_1_ and *T*_2_ in a top-down approach. For every mapping $n\in T_{2}$ on $m\in T_{1}$, it compares unmatched children of *n* and *m* to find missed mappings. A distance matrix $M^{i\times j}$ is created with $M_{i,j}$ being the ratio of the number of differing attributes to the total number of attributes between the *i*th child of *n* and the *j*th child of *m* or 0 if both nodes do not have any attributes. We assign ∞ to elements $M_{i,j}$ if the corresponding nodes already have a mapping or if they do not share the same tag name. The algorithm evaluates the matrix greedily and adds new mappings up to a maximum distance of 0.9. Thus, nodes which have nothing in common will not be connected.

### 2.3 Post-processing the resulting mapping

Additional mapping rules capture the domain characteristics of the processed data. Following the current specifications for SBML and CellML, we prohibit certain changes in the hierarchical tree of document nodes. Specifically, we treat parts of the model as atomic constructs for which we define restrictions on possible network operations.

In SBML models, e.g. listOf-nodes must not change their parents. That means, if a listOfModifiers of *T*_1_ is mapped onto a listOfModifiers of *T*_2_ but their parents are not linked, then we drop this mapping. Similarly, nodes with tag names speciesReference, trigger, eventAssignment, modifierSpeciesReference, delay and priority are glued to their respective parents. If the parents in the corresponding tree are not connected, which means their networks in the XML documents differ, we remove the mapping from the set of operations. In CellML models, nodes with tag names variable and reaction are glued to their components.

Obviously, these rules expand the set of operations in the delta later on, but we are willing to trade some minimality to increase the significance of produced deltas. This step is a major reason why our algorithm outperforms standard XML diff algorithms.

### 2.4 Computing the delta

A delta is a set of operations on entities (nodes or attributes, respectively) necessary to transform one document into another. We distinguish the following four types of operations which apply on entities of the corresponding XML tree:

*insert* if an entity is present in *T*_2_ but absent in *T*_1_
*delete* if an entity is present in *T*_1_ but absent in *T*_2_
*move* if a node is present in both documents, but either (i) the parents in the corresponding trees are not connected or (ii) the parents are connected, but the sequence of their siblings has changed
*update* if the value of an attribute, a text node’s content or the tag name of a node was modified

While the set of move operations may only contain document nodes, the set of updates in general only consists of operations on attribute values. There is a single exception: the root nodes of both documents are always mapped onto each other. Therefore, we must include an operation which updates the tag name of nodes. However, we only support this operation for root nodes. Thus, internal document nodes will never occur in the set of updates and we will neglect this special case in the following.

After the mapping, we distinguish two types of nodes: mapped nodes and unmapped nodes. Unmapped nodes $n\in T_{1}\cup T_{2}$ are nodes for which the algorithm could not find a matching node in the opposite tree. These nodes and their attributes correspond to either inserts or deletes, depending on their origin (*T*_2_ or *T*_1_, respectively). In contrast, mapped nodes are nodes for which the algorithm did find a matching node in the opposite tree. If the parents of such a mapping of $n\in T_{2}$ onto $m\in T_{1}$ are not connected, or if the sequence among their siblings has changed, then these nodes are included in the set of moves. Moreover, for each attribute a∈attributes(n)∪attributes(m):

- if a∉attributes(m) then *a* is included into the set of deletes
- if a∉attributes(n) then *a* is included into the set of inserts
- if value(m,a)≠value(n,a) the attribute is included into the set of updates

All other cases (nodes are mapped and occur at the same position in both trees; attribute values of mapped nodes are equal) do not call for an operation to transform *T*_1_ into *T*_2_ and are therefore not included in the delta.

### 2.5 Translation into machine readable XML

The resulting delta is then encoded in an XML document consisting of the four sections *deletes*, *inserts*, *moves* and *updates.* These sections contain three types of nodes:

- Nodes with a tag name node, describing operations on nodes
- Nodes with a tag name attribute, describing operations on attribute values
- Nodes with a tag name text, describing operations on text nodes

All these nodes have to carry a unique id attribute and, if available, must contain identifiers oldPath and newPath to unambiguously point to the corresponding nodes in *T*_1_ and *T*_2_, respectively. These identifiers are XPath (www.w3.org/TR/xpath/) expressions, a language defined by the World Wide Web Consortium (W3C), to identify nodes in an XML document. In addition, node nodes may also contain the attributes:

- oldParent and newParent (XPath expressions), identifying the parents of the corresponding nodes
- oldChildNo and newChildNo (Integers), defining the position among their siblings, in order to encode *moves*
- oldTag and newTag (Strings), specifying the tag name of the corresponding nodes

Furthermore, attribute nodes may have three additional attributes:

- name, defining the name of the corresponding attribute
- oldValue and newValue, specifying the value of that attribute in *T*_1_ and *T*_2_, respectively

The generated delta is complete and, thus, it is invertible. That means, it contains all information necessary to transform *T*_1_ into *T*_2_, but it can also be used to obtain *T*_1_ given *T*_2_. Figure 3c shows an example.

### 2.6 Translation into human readable formats

To support the readability, we currently export two different formats: (i) A text-based report and (ii) a graphical representation. The text-based report is a list of differences between the corresponding files. It contains all modified entities relevant for the biological model, such as parameters, species and reactions, and it contains the specific changes. The report can be generated in HTML, ReStructuredText or Markdown. Markdown and ReStructuredText are easy-to-read plain-text markup languages, specifically designed to ensure a straightforward conversion to other markup languages, such as (X)HTML, doc(x), ODF or LATEX.

Moreover, standard formats usually allow for the inclusion of information about the reaction network encoded in the computational model. If models contain such information, we extract the reaction network of *T*_1_ and translate it into an internal graph representation. We then obtain the reaction network of *T*_2_ and put it as an overlay on top of the network of *T*_1_, using the previously computed mapping of entities in *T*_1_ and *T*_2_. Subsequently, the graph is evaluated: We check whether nodes and edges originate from one or both documents and analyse what has changed in the corresponding tree nodes. To export this graph, we developed translators that convert the internal graph representation to exchangeable graph formats, such as GraphML (graphml.graphdrawing.org/) or Dot (graphviz.org/content/dot-language). These graphs can then easily be used in end-user applications.

## 3 Results

Models are continuously modified. Consequently, new versions of a model are regularly being generated. We observe three major steps in model development that result in new versions: during the design phase of models; later on during curation and error correction and with updates of the format specification. Here, we present an algorithm to detect and communicate differences between these co-existing versions of an SBML or CellML model. We implemented this algorithm in a software library, BiVeS. Together, our work helps to identify and characterize the changes and thereby contributes to the documentation of a model’s history.

### 

#### 

##### BiVeS detects the differences between model versions

**Fig. 2.**
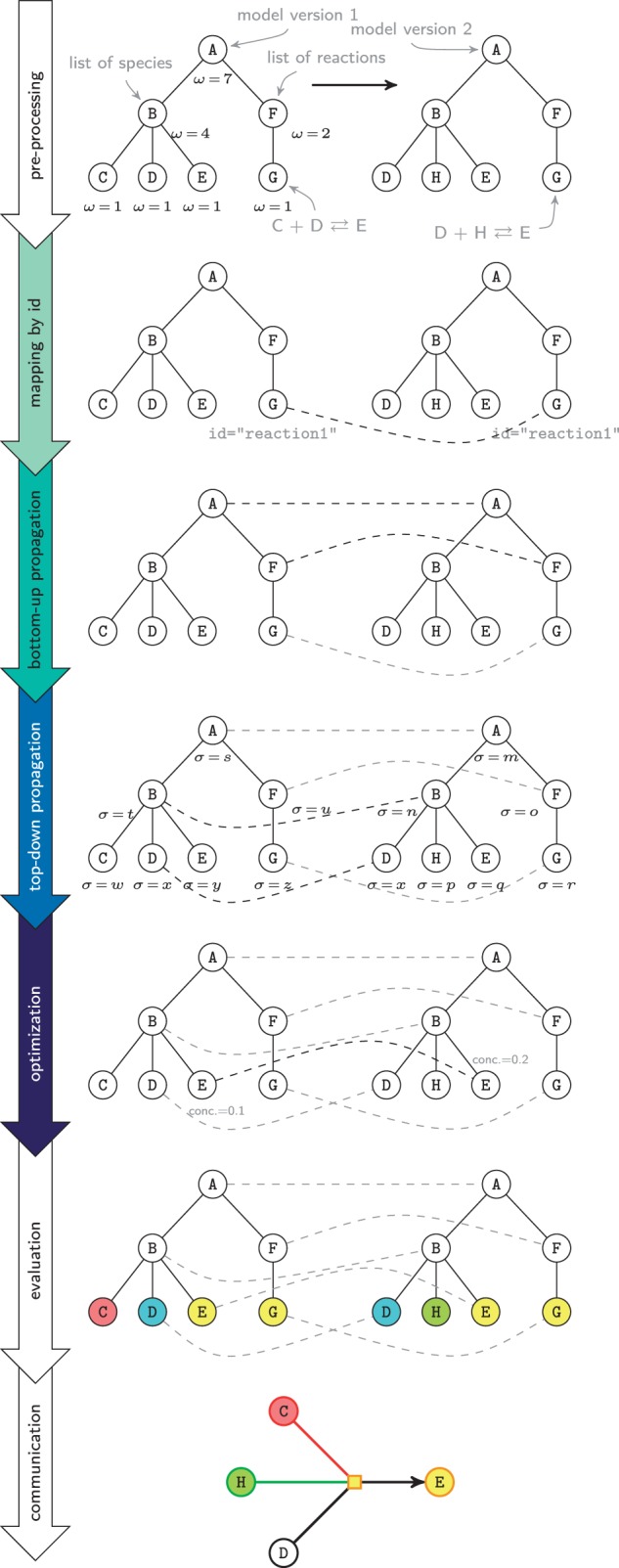
**Schematic of the mapping procedure.** The procedure to communicate the differences between two versions of a model (row one) to the user (row seven) is shown. Nodes A–H represent single entities in the model documents. Dashed lines indicate mappings between the nodes. The values of σ and ω represent signatures and weights of nodes. They are calculated during pre-processing. The different colours of the nodes indicate modifications: updates are yellow, inserts are green and deletes are red. In the evaluation step, moves are blue

**Fig. 3.**
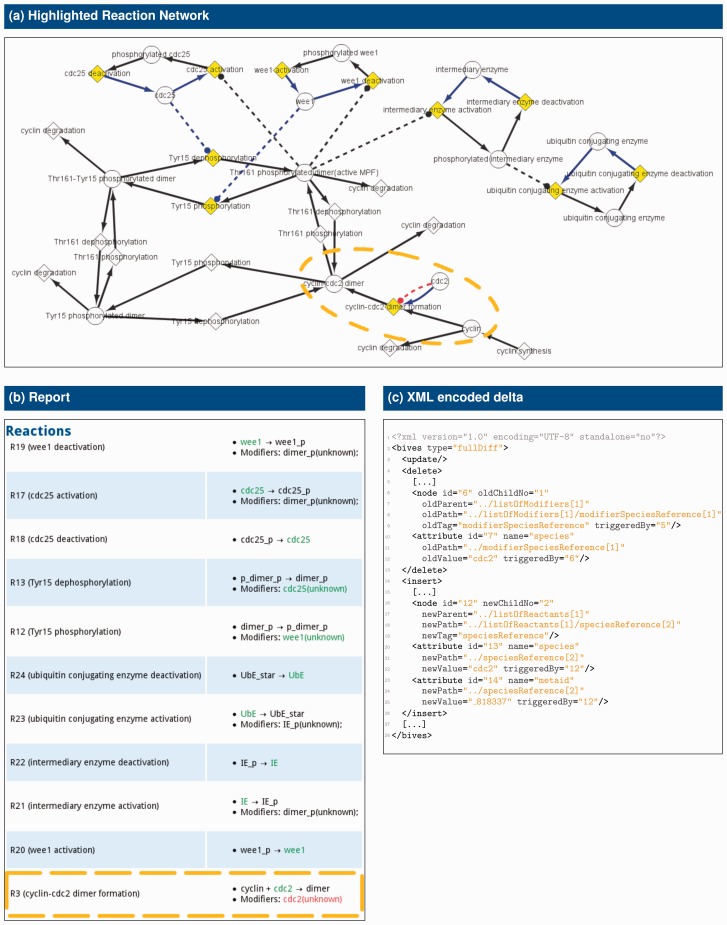
**Outputs as generated by BiVeS and available from BudHat.** All three figures show the differences between versions June 2007 and November 2013 of model BIOMD0000000107 (cf. Fig. 1). The reaction network (**a**) and the report (**b**) present the differences in a human readable format. The XML encoded delta (**c**) allows for further processing by computers. The modifications described in Figure 1 are highlighted in orange. In the highlighted reaction network (a), deletes are coloured in red, while inserts are blue and updates are yellow

Figure 2 exemplifies the method, showing two versions of a minimalist model, following the SBML structure. Here, the reaction C+D⇄E (left) is updated to D+H⇄E (right). First, the model files are transformed into internal tree representations and prepared for the subsequent mapping procedure (row one, *pre-processing*). The weights *ω* of nodes in the tree are computed according to the size of the corresponding subtrees. For example, the subtree rooted in B is larger than the subtree rooted in F and, thus, B’s weight is greater than F’s (namely *ω* = 4 and *ω* = 2, respectively). The mapping procedure starts in row two of Figure 2 with a *mapping by id.* Since the id attribute plays a key role and many elements do carry id attributes, the algorithm typically finds a large number of mappings at this early stage. In our example, only the identifiers of the G-nodes are identical (id=’reaction1’) and thus only a single connection is found (for demonstration purposes, we assume that the D-nodes do not carry id attributes). The *mapping by id* phase is followed by a *bottom-up propagation* (row three), which makes use of the parent-child relation of nodes in the trees: For nodes that are mapped already, there is a good chance that their parents also stem from each other. In the example, the mapping of the G-nodes is propagated towards the roots of the trees and the A-F-G-paths in both model versions are mapped. Afterwards, the algorithm tries to map subtrees with an equal signature (row four, *top-down propagation*). The signatures *σ*, which are computed in the *pre-processing* step, uniquely identify the subtrees. Here, only the signatures of the D-nodes are equal (σ=x), which is why D is the only candidate for a mapping. Since the D-nodes originate from each other, as well as the A-nodes do, a mapping of the B-nodes is added. Following the propagation phases, the algorithm tries to connect unmapped children of mapped nodes (row five, *optimization*). In our example, only the B-nodes have unmapped children: Nodes C and E in version 1 and nodes E and H in version 2 do not yet have partners. To find a mapping of these children, a 2 × 2 distance matrix is created. The elements in this matrix represent differences between the attributes of the corresponding nodes. The E-nodes only differ in the value of the concentration attribute. Changing the value of one single attribute in a species is a minor update and, thus, the nodes’ distance is very small. In contrast, the nodes C, E and H do not have anything in common. Consequently, the E-nodes will be mapped, while C and H remain unmapped. Finally, the resulting mapping is analysed (row six, *evaluation*). For example, the algorithm detects that C was deleted, D was inserted and E was modified in version 2. The difference graph, as obtained when interpreting the results of the *evaluation* step, is shown on the bottom of Figure 2. Particular means of communicating the differences are described with Figure 3. The Supplementary Material contains a more detailed, real-life showcase for difference detection with BiVeS.

##### BiVeS communicates the differences

BiVeS exports the difference graph in several output formats, including computer-digestible XML code and a graphical representation.

One type of output are XML-encoded, machine readable deltas, which describe the difference between two versions of a model (see Section 2.4). A remarkable feature of these deltas is their completeness. They can be inverted and composed (Marian *et* *al.*, 2001). That means, given one model version and the delta, the opposite version can be retrieved (Saffrey and Orton, 2009).

Another major feature is the translation of the delta into human readable formats (refer to the *communication* step in Fig. 2). BiVeS, for example, summarizes the model-related changes in a text-based report. This type of output is ideally suited to be integrated in other tools. Specifically, the report is encoded in MarkDown, ReStructuredText or HTML. MarkDown and ReStructuredText are themselves already easy to read and can be converted to common markup languages. The report in HTML format is generated for convenience, e.g. to instantly display the changes on a web page. Figure 3b shows a sample report. Another notable feature is the encoding of differences between two versions of a model in standard graph representations enabling a subsequent visualization. While BiVeS is itself not able to produce rendered graphical output, it exports different graphical notations, including GraphML, Dot or JSON (json.org). Armed with this, it is effortlessly possible to produce visualizations, as implemented in the demonstrator BudHat.

##### BudHat demonstrates the advantages of our algorithm

As a proof of concept, we implemented a web-based interface BudHat (budhat.sems.uni-rostock.de), which uses BiVeS to compare versions of a computational model. BudHat contains a rudimentary user management and stores models in a database back-end. It calls BiVeS for the comparison and displays the obtained results in the web browser. The different visualizations that are possible in BudHat are shown in Figure 3. All figures show the difference between versions 2007-06-05 and 2013-11-03 of model BIOMD0000000107 in the BioModels database.

More specifically, BudHat provides access to (i) the reaction network highlighting the changes, as shown in Figure 3a; (ii) the HTML report of the changes, as shown in Figure 3b and (iii) the delta encoded in XML, as shown in Figure 3c. Delta and report are directly passed to the web interface. But as stated above, BiVeS exports the graph representing the reaction network in an exchangeable format (in this case it is GraphML or JSON). Therefore, BudHat uses either CytoscapeWeb (Lopes *et* *al.*, 2010) or CytoscapeJS (cytoscape.github.io/cytoscape.js) to display the highlighted reaction network. From Figure 3a, it is easy to see that the role of cdc2 in reaction cyclin-cdc2 dimer formation has changed. In the former version, cdc2 modified this reaction, but this modification was deleted (deletion is highlighted by the red edge). Instead, in the updated version, cdc2 is one of the reactants for this reaction (insertion is indicated by the blue edge). Since this modification changed the reaction, the node representing the reaction is coloured in yellow. This approach makes it much easier to understand the differences, compared to a pure textual diff. Already for this small example, it would be much more effort to see and understand what happened to a model from the sources or from the 1559 lines of output reported by Unix’ diff.

## 4 Discussion

Reproducibility of model-based scientific results has gained increasing attention (Casadevall and Fang, 2010; Gentleman, 2005; Laine *et* *al.*, 2007; Mesirov, 2010; Peng, 2011; Sandve *et al.*, 2013; Waltemath *et* *al.*, 2011, 2013b). Indeed, the ability to reproduce results is a basic requirement for the advance of science (King *et* *al.*, 2011). However, the reuse of models requires the accessibility and comparability of models and their versions. Model provenance and version control enable the widespread use and application of models, saving time and efforts during development. Model repositories have been working towards this goal for the past decade and provide access to computational models described in scientific publications. Support for version control, however, is still limited. Existing implementations rely on standard version control systems and do not consider the specific requirements of modelling in the domain of computational biology (Waltemath *et* *al.*, 2013a). Model repositories, specifically the BioModels database and the Physiome Model Repository, could benefit from integrating BiVeS with their solutions for version control. Such a combined system stores model versions and detects the differences between them. In addition, it offers support for understanding these changes and filtering them according to the users’ preferences, as discussed in the following.

### 

#### 

##### BiVeS improves difference detection for your model versions

Standard formats describing computational models in biology are based on XML. Changes in versions of these models are typically computed with Unix’ diff, which performs badly on XML documents because it uses a line-based algorithm (Myers, 1986). BiVeS, on the other hand, is designed to respect the characteristics of XML documents and to produce meaningful deltas. Its major advantages over existing solutions for biological models are as follows: (i) it recognizes the models’ hierarchical structures; (ii) it ignores white spaces, such as indentation, which generally do not affect the model’s behaviour and (iii) it ignores the specific order of attributes in an entity. Additional post-processing rules capture the domain characteristics of the processed data and increase the significance of produced deltas. Currently, these rules are static. However, we consider introducing adjustable rules, which can be modified and extended. In the Supplementary Material, we compare the BiVeS diff and standard Unix’ diff. We also discuss the dependencies of modifications, explain the concepts of direct and implicated operations and provide statistics about model changes in the BioModels database and in the Physiome Model Repository (see Supplementary Figs A and B in the Supplementary Material). From the data, we assume that we can further improve the *mapping by ID* step, described in Section 2.2. Currently, we just map entities having the same identifiers. In future versions, we would like to also map entities based on their ontological similarity.

##### BiVeS helps grasping the changes

Our algorithm filters the identified differences and drops all but biologically and mathematically relevant modifications. We are currently working on refining these filters using an ontology for differences. We envision that this ontology, together with tools for semi-automatic annotation, will help reduce the number of displayed changes to the ones that are meaningful or requested by the user (Waltemath *et* *al.*, 2013a). BiVeS produces reports and graphical representations of changes using open formats such as GraphML, HTML or Markdown and thereby helps to communicate the changes. For example, Figure 3a shows that the graphical representation supports users in exploring the changes affecting the biological network. Additionally, a comprehensive list of changes is compiled into a human readable report, as shown in Figure 3b. Reports are particularly suitable for people interested in the details of mathematical changes. BiVeS’ outputs can of course be used by other tools for further processing of results. Current limitations with respect to output formats are missing support for the SBGN format and suitable graphical representations of models that do not specify a reaction network.

##### You can easily integrate BiVeS with your tools

BiVeS can be used in three different ways: first, the BiVeS Java library provides a smart API for comparison of model versions. The differences can then be obtained in various formats, as described earlier. This API is, for instance, used by our open source tool BudHat, providing plenty of example code. Second, BiVeS is available as a web service to facilitate the integration with non-Java applications. The corresponding package can be installed on Java-based web servers, such as Apache Tomcat (tomcat.apache.org). The Functional Curation (chaste.cs.ox.ac.uk/FunctionalCuration) project of Chaste (Cooper *et* *al.*, 2011), for example, uses the BiVeS web service to track the evolution of models uploaded to their system. Third, the library is shipped with a main class and, therefore, it can be executed on a command line. The data management platform SEEK (Wolstencroft *et* *al.*, 2015), for example, implemented support for model version control calling BiVeS on a separate command line. The web site at sems.uni-rostock.de/bives offers further information about the three implementations, including examples, how-tos, the source code and binaries of our tools. BiVeS currently supports SBML and CellML, but it could also be extended towards other XML-based model exchange formats such as NeuroML (Gleeson *et* *al.*, 2010) or PharmML (Swat *et* *al.*, 2015). Moreover, BiVeS could improve version control of simulation descriptions [e.g. differences between two simulation setups encoded in the Simulation Experiment Description Markup Language (SED-ML), Waltemath *et* *al.*, 2011].

In summary, BiVeS improves the detection of differences between versions of models in SBML or CellML format. Returning to the seven W-questions from the introduction, BiVeS contributes to the *What* and *How* as defined in (Ram and Liu, 2010). The *What* refers to content-related events, such as modifications of parameter values in the model, and non-content-related events, such as the upgrade to a new SBML version. In addition, BiVeS tells you *How* the *What* has changed. There is scope for further extensions to provide hypotheses for the *Why*.

## Supplementary Material

Supplementary Data
