# Supplementary material for: An algorithm to detect and communicate the differences in computational models describing biological systems
Source: Bioinformatics. 2015 Oct 21;32(4):563–70. doi: 10.1093/bioinformatics/btv484 (PMC4743622; doi:10.1093/bioinformatics/btv484)
Supplement: Supplementary Data [file supp_32_4_563__index.html]

An algorithm to detect and communicate the differences in computational models describing biological systems — An algorithm to detect and communicate the differences in computational models describing biological systems — Supplementary Data 

# An algorithm to detect and communicate the differences in computational models describing biological systems

## Supplementary Data

files

- Supplementary Data - zip file
