# Supplementary material for: An algorithm to detect and communicate the differences in computational models describing biological systems
Source: Bioinformatics. 2015 Oct 21;32(4):563–70. doi: 10.1093/bioinformatics/btv484 (PMC4743622; doi:10.1093/bioinformatics/btv484)
Supplement: Supplementary Data [file supp_btv484_suppl_data.zip › S0.pdf]

Supplementary Information

An algorithm to detect and communicate the differences in computational models describing biological systems

Martin Scharm [1], Olaf Wolkenhauer [1,2] and Dagmar Waltemath [1]

[1]Department of Systems Biology and Bioinformatics, University of Rostock, Rostock, Germany

[2]Stellenbosch Institute for Advanced Study, Wallenberg Research Centre at Stellenbosch University, Stellenbosch, South Africa

1 BiVeS improves difference detection for your model versions.

Standard formats describing computational models in biology are based on XML. Changes in versions of these models are typically computed with Unix' diff, which performs badly on XML documents because it uses a line based algorithm (Myers, 1986). BiVeS, on the other hand, is designed to respect the characteristics of XML documents and to produce meaningful deltas. For example, it recognises the models' hierarchical structures (Figure C). Furthermore, it ignores white spaces, such as indentation, which generally do not affect the model's behaviour. Finally, it ignores the specific order of attributes in an entity. Figure B compares the deltas as obtained by Unix' diff and BiVeS. To generate the data, we compared all versions of publicly available models from the BioModels database (top row) and the Physiome Model Repository (bottom row). The boxplots on the left hand side of Figure B show that BiVeS needs fewer operations than Unix' diff to transfer one version of a model into another (avg. number of operations needed by BiVeS —Unix' diff: Physiome Model Repository 206.900—679.800; BioModels Database 222.119—1515.479). The scatter plots on the right hand side of Figure B confirm this trend.
One explanation, why BiVeS needs less operations than Unix' diff, is that BiVeS recognises moving entities. For example, if the sequence of reactions changes, Unix' diff merely detects insert and delete operations. Lets assume the reaction from Figure C gets moved to a different location in the document. Unix' diff would report up to 21 inserts and 21 deletes. In contrast, BiVeS recognises this modification as a single move operation. Indeed, move operations occur often, as shown in Figure A. As the prominent blue band indicates, most version updates (83.7%) in the Physiome Model Repository include at least one move operation. Moreover, 57.9% of these updates contain more than 5 moves.
In addition, we studied the ratio of direct and implicated operations. Deleting an entity, for instance, is a direct operation. The subsequent deletion of its attributes is triggered and, thus, an implicated operation. In both datasets, the number of direct operations is higher

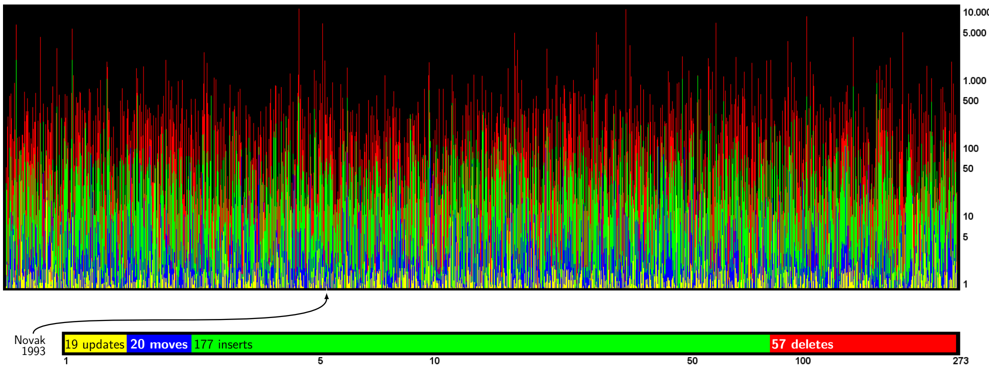

Figure A: Nature of changes in the Physiome Model Repository. Each bar corresponds to a version update. The y-axis shows the size of the delta on a logarithmic scale. The colours of each bar show the composition of the deltas: Deletes in red; inserts in green; moves in blue; and updates in yellow. The number of operations of each bar is plotted on a linear scale (as demonstrated for one update of the Novak 1993 model, from 5. July 2010). The prominent blue band represents the large number of move operations identified by BiVeS. All data is taken from Physiome Model Repository, all releases till June 2015. The plot includes all transitions between versions with at least 10 operations.

than the number of operations they implicate (Figure B, bottom boxplots). This suggests that only a few modifications entail implicated operations.

2 Exemplifying difference detection for SBML models.

We exemplify the advantages of our method using a reaction extracted from two different releases of model BIOMD0000000107 in the BioModels database. Figure C shows the SBML code of versions V_1 (June 2007, release 8) and V_2 (November 2013, release 26). BiVeS can now be used to compare these versions. Before starting the mapping procedure, it takes both model files and pre-processes them (see Section 2.1 in the main document). For every node a signature and a weight are calculated. The signature uniquely identifies the subtree rooted in this node. A node's weight corresponds to the size of its subtree. The internal representations of the models can then be mapped (see Section 2.2 in the main document). The algorithm first connects all nodes having an equal value of the id attribute (*mapping by id*). In this example, only the reaction nodes in both model versions carry an identical id attribute. Consequently, this step adds one mapping. Mapped nodes then trigger a mapping of the parent nodes (*bottom-up propagation*). In this case, the mapping of the `reaction` nodes entails a mapping of the `listOfReactions` nodes in both documents. Subsequently, BiVeS evaluates the previously calculated signatures and maps equal subtrees. Since nothing has changed in the subtrees rooted in the `kineticLaw`

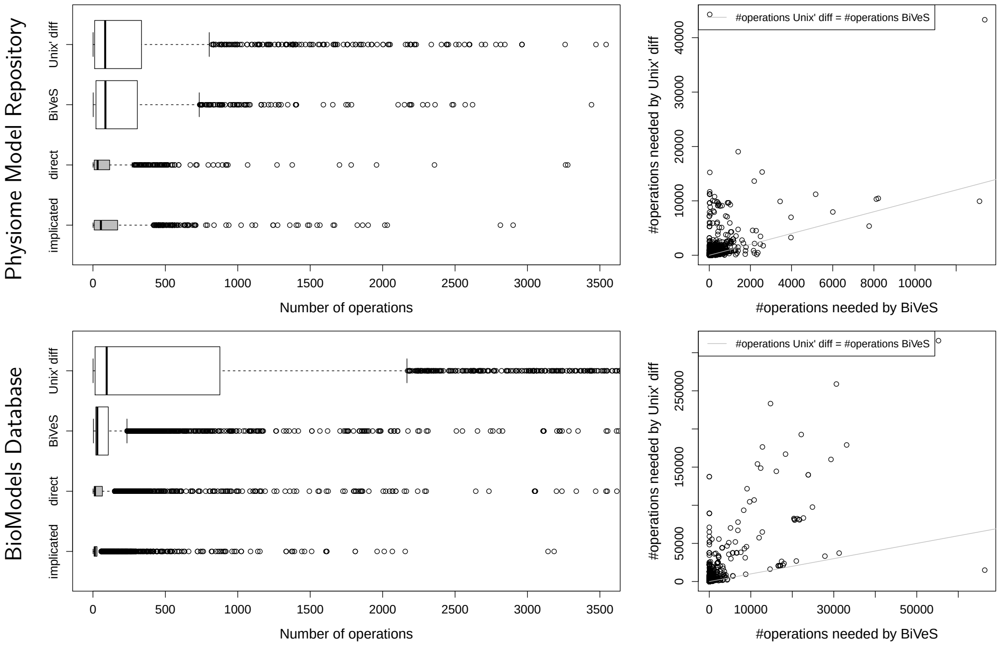

Figure B: Comparison of operations needed by Unix' diff and BiVeS. The top row displays results obtained from models of the Physiome Model Repository (1730 publicly available model transitions) and the bottom row displays results obtained from models of the BioModels database (7594 publicly available model transitions). On the left hand side bloxplots show the frequency of number of operations needed by Unix' diff and BiVeS. In addition, the operations as reported by BiVeS are divided into direct and implicated operations. On the right hand side, a scatterplot visualises the number of operations needed by Unix' diff and BiVeS for particular files.

nodes, they are mapped onto each other. BiVeS then searches for mapped nodes which have children without a mapping (*optimisation*). Here, the node `reaction` is mapped, but its children are not, which triggers the creation of a distance matrix. Since the `annotation` nodes in both trees are equal the corresponding matrix element is 0. Same applies to the `listOfReactants` and `listOfProducts` nodes. All other elements in this matrix are ∞. Therefore, BiVeS adds three additional mappings. The optimisation phase recursively applies this procedure to all the children. First, it continues with a recursive mapping of the nodes in the `annotation` subtree. It stops at the `rdf:li` nodes, because these nodes have nothing in common but their tag names. The algorithm will not connect them. Second, BiVeS finds three unmapped children below the `listOfReactants` nodes (the `speciesReference` nodes representing Cyclin in V_1 and Cyclin/Cdc2 in V_2). While the computed distance between Cyclin nodes is $\frac{1}{3}$ (the value of one attribute differs), the distance between the Cyclin and Cdc2 nodes is 1 (no matching attributes). Therefore, a mapping between the `speciesReference` nodes of the Cyclins is introduced. Third, a mapping between the `speciesReference` nodes (distance: $\frac{1}{3}$) below the `listOfProducts` nodes is detected. Eventually, the algorithm was able to find a mapping for most of the nodes. The unmapped nodes `rdf:li` and `speciesReference` (for species Cdc2) in V_2 correspond to inserts, and the unmapped node `rdf:li` as well as the subtree `listOfModifiers` correspond to deletes. An extract of the delta produced from the sample versions in Figure C is given in Figure 3c of the main document (XML is attached as S1 and S2). The shown delta describes the move of the subtree rooted at the fifth node below the first `reaction` (first node with a tag name of `kineticLaw`). This subtree was moved and became the fourth child of the same `reaction` node in version two (ll. 5).
The presented example is fairly small and straightforward. Yet it demonstrates how BiVeS easily outperforms Unix' diff. While BiVeS detects five deletes, eight inserts and one move, Unix' diff needs 19 deletes and 16 inserts. Apparently, Unix' diff is unable to meaningfully explain the modifications. The output of both tools can be found in the supplementary material.

References

Myers, E. W. (1986). An o(nd) difference algorithm and its variations. *Algorithmica*, **1**, 251–266.

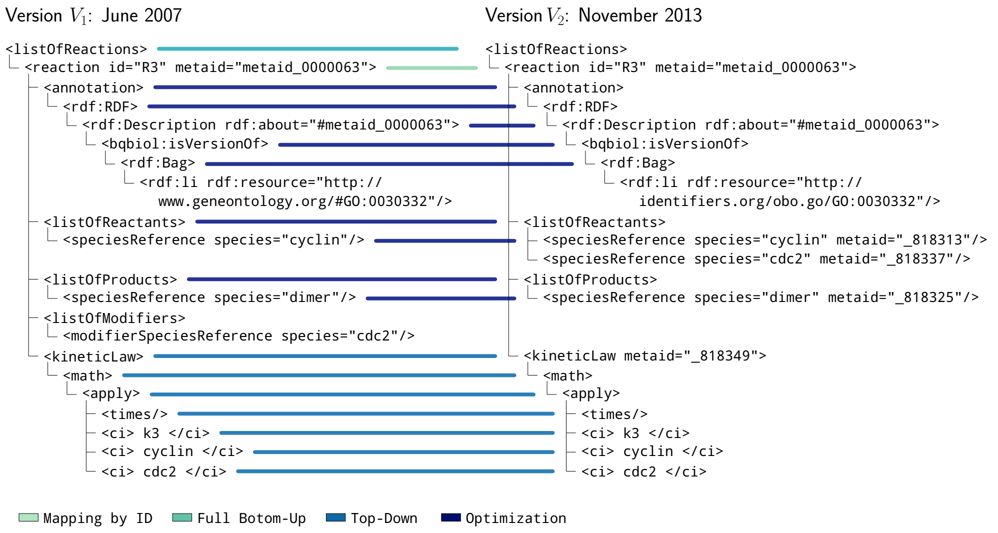

Figure C: Example of a computed mapping. The figure shows the two versions V_1 and V_2 of the highlighted reaction from Figure 3 in the main document. The differently coloured lines visualise the mapping of entities obtained when applying the four steps as described in Figure 2 of the main document. Gaps between at rows on the left indicate inserted items, gaps on the right denote deleted items.
